# Marine reserves indirectly affect fine‐scale habitat associations, but not overall densities, of small benthic fishes

**DOI:** 10.1002/ece3.2406

**Published:** 2016-08-29

**Authors:** Adam N. H. Smith, Marti J. Anderson

**Affiliations:** ^1^ Institute of Natural and Mathematical SciencesMassey UniversityAucklandNew Zealand; ^2^ New Zealand Institute for Advanced StudyMassey UniversityAucklandNew Zealand

**Keywords:** Bayesian hierarchical modeling, behavior, habitat association, indirect effects, multivariate generalized linear mixed models, no‐take marine reserves, risk effects, small benthic fishes, temperate rocky reef, Tripterygiidae

## Abstract

Many large, fishery‐targeted predatory species have attained very high relative densities as a direct result of protection by no‐take marine reserves. Indirect effects, via interactions with targeted species, may also occur for species that are not themselves targeted by fishing. In some temperate rocky reef ecosystems, indirect effects have caused profound changes in community structure, notably the restoration of predator–urchin–macroalgae trophic cascades. Yet, indirect effects on small benthic reef fishes remain poorly understood, perhaps because of behavioral associations with complex, refuge‐providing habitats. Few, if any, studies have evaluated any potential effects of marine reserves on habitat associations in small benthic fishes. We surveyed densities of small benthic fishes, including some endemic species of triplefin (Tripterygiidae), along with fine‐scale habitat features in kelp forests on rocky reefs in and around multiple marine reserves in northern New Zealand over 3 years. Bayesian generalized linear mixed models were used to evaluate evidence for (1) main effects of marine reserve protection, (2) associations with habitat gradients, including complexity, and (3) differences in habitat associations inside versus outside reserves. No evidence of overall main effects of marine reserves on species richness or densities of fishes was found. Both richness and densities showed strong associations with gradients in habitat features, particularly habitat complexity. In addition, some species exhibited reserve‐by‐habitat interactions, having different associations with habitat gradients inside versus outside marine reserves. Two species (*Ruanoho whero* and *Forsterygion flavonigrum*) showed stronger positive associations with habitat complexity inside reserves. These results are consistent with the presence of a behavioral risk effect, whereby prey fishes are more strongly attracted to habitats that provide refuge from predation in areas where predators are more abundant. This work highlights the importance of habitat structure and the potential for fishing to affect behavioral interactions and the interspecific dynamic attributes of community structure beyond simple predator–prey consumption and archetypal trophic cascades.

## 1 Introduction

No‐take marine reserves are increasingly being used to protect marine ecosystems from local‐scale anthropogenic impacts of fishing. Following the implementation of no‐take status, species that are targeted by fishing often increase in average size, density, and biomass within reserves (e.g., Lester et al., 2009). The magnitude of the effects of any particular reserve on any particular species is highly variable. Some general patterns have emerged, however; heavily fished, large‐bodied species occupying high trophic levels (Guidetti et al., 2014; Molloy, McLean, & Côté, 2009) that occur in larger, older, isolated, well‐enforced reserves (Edgar et al., 2014) tend to show the greatest positive responses to protection.

Marine reserves are often promoted as management tools for conserving not only those species targeted by fishing, but biodiversity more generally (e.g., Agardy, 1994), motivating evaluations of the effects of reserves on broader marine communities. Where fishing includes significant bycatch or causes damage to habitats, such as bottom trawling in soft sediments, nontargeted species and communities may be directly affected by fishing (Jennings & Kaiser, 1998), and hence may benefit directly from protection (Game et al., 2009). However, on coastal reefs, where more selective fishing methods are generally used, effects on the broader community are most likely to occur indirectly through interactions with protected, fishery‐targeted species (Babcock et al., 2010; Claudet, Guidetti, Mouillot, Shears, & Micheli, 2011).

Re‐established predator–prey relationships have, in some reserves, caused profound changes in community structure through trophic cascades. For example, in many reserves established on temperate rocky reefs, increases in large predatory mammals, fish, or invertebrates, such as lobster, have reduced the abundance of herbivorous sea urchins, allowing regeneration of macroalgal forests in areas previously dominated by urchin‐grazed “barrens” habitat (e.g., Babcock, Kelly, Shears, Walker, & Willis, 1999; Ling et al., 2015). Yet, generalities concerning the variable indirect effects of marine reserves remain elusive, as they depend on the environmental and ecological context (Salomon et al., 2010; Shears, Babcock, & Salomon, 2008), the strength of the reserve effect for species targeted by fishing, and the strength and nature of relationships between targeted and nontargeted species (Jennings & Kaiser, 1998; Micheli et al., 2005).

Nontargeted small benthic fishes are a functionally important and conspicuous component of the biodiversity on many coastal reefs. It may be expected that some of these species would show indirect effects of reserves where large piscivorous fishes have responded positively to protection (Micheli, Halpern, Botsford, & Warner, 2004). Many experimental studies have found that predators can indeed have a role in controlling the abundance of prey fishes, particularly on coral reefs (e.g., Carr & Hixon, 1995; Hixon & Jones, 2005). Yet, empirical evidence for such indirect effects of reserves has been mixed; some studies have found patterns consistent with indirect effects (e.g., Edgar & Stuart‐Smith, 2009; Graham, Evans, & Russ, 2003; McClanahan, Muthiga, Kamukuru, Machano, & Kiambo, 1999) but many have not (e.g., Jennings, Grandcourt, & Polunin, 1995; Langlois, Harvey, & Meeuwig, 2012; Russ & Alcala, 1998; Tetreault & Ambrose, 2007). Meta‐analyses too have provided mixed results: the responses of fish species not targeted by fishing to marine reserve protection show a lot of variation but are apparently, on average, unaffected by reserves (Claudet et al., 2010; Micheli et al., 2004; Mosquera, Côté, Jennings, & Reynolds, 2000).

Many fishes are generalist predators and are able to consume a wide variety of prey (Jennings & Kaiser, 1998), though will generally target small prey (e.g., around 5% of their own body weight, Edgar & Shaw, 1995). Prey species can also alter their behavior or choice of habitat when predators are present, in order to minimize the risk of predation (Creel & Christianson, 2008; Lima & Dill, 1990; Preisser, Bolnick, & Benard, 2005). Behaviorally mediated indirect interactions, or “risk effects,” are increasingly being recognized as important in ecology, perhaps even more than density‐mediated effects of predation (Creel & Christianson, 2008; Peacor & Werner, 2001; Schmitz, Krivan, & Ovadia, 2004), even in the marine environment (Dill, Heithaus, & Walters, 2003; Grabowski, 2004; Heithaus, Frid, Wirsing, & Worm, 2008). Yet, evaluations of indirect effects in marine reserves have focused on changes in the abundance of prey, while little consideration has been given to evaluating potential risk effects.

**Figure 1:**
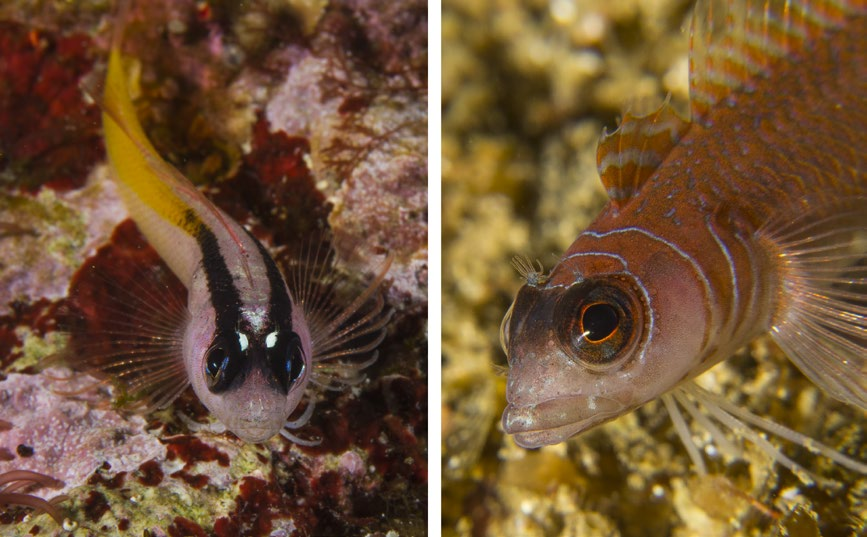
Two species of Tripterygiidae, *Forsterygion flavonigrum* (left), and *Ruanoho whero* (right), both endemic to New Zealand. Photographs by Paul Caiger

In northeastern New Zealand (NZ), the restoration of high densities of predatory fish inside marine reserves is well documented. In particular, relative densities of large snapper (*Pagrus auratus*, Sparidae) are around 13 times greater inside versus outside reserves in this region, on average (Smith, Anderson, Millar, & Willis, 2014; Willis, Millar, & Babcock, 2003). Snapper are generalist, opportunistic predators of primarily invertebrates and fishes (Godfriaux, 1969), and have been shown to play a role in indirect effects of reserves, including a predator–urchin–kelp trophic cascade on rocky reefs (Babcock et al., 1999; Shears & Babcock, 2003; Shears et al., 2008). Willis and Anderson (2003) reported lower densities of small benthic reef fishes, including a highly diverse group of endemic triplefins (family Tripterygiidae, Fig. 1), within a single reserve, potentially due to increased predation. Spatial and temporal replication in that study was limited, however: Sets of replicate sampling units were taken from just two sites inside and two sites outside one reserve at a single time. Triplefins are strongly associated with small‐scale habitat features that provide refuge from predators (Feary & Clements, 2006; Syms, 1995; Wellenreuther, Barrett, & Clements, 2007), and the maintenance through time of the density of at least one species (*Forsterygion varium*) appears to depend on the availability of such features (Connell & Jones, 1991).

In light of these studies, it is reasonable to expect an interaction between the effects of reserves (where predators are more abundant) and the availability of refuges from predation (i.e., habitat complexity) on densities of small benthic fishes. We consider that at least two mechanisms could contribute to such an interaction. Firstly, small fishes are consumed at greater rates inside marine reserves where predators are in higher densities, with small fishes living in less complex habitats being more vulnerable to predation (Connell & Jones, 1991). Secondly, small fishes may perceive high densities of predators inside reserves and respond with a risk effect whereby they are more strongly attracted to complex refuge‐providing habitats. Both mechanisms described above would lead to the relationship between densities of small fishes and habitat complexity being stronger inside versus outside marine reserves.

**Figure 2:**
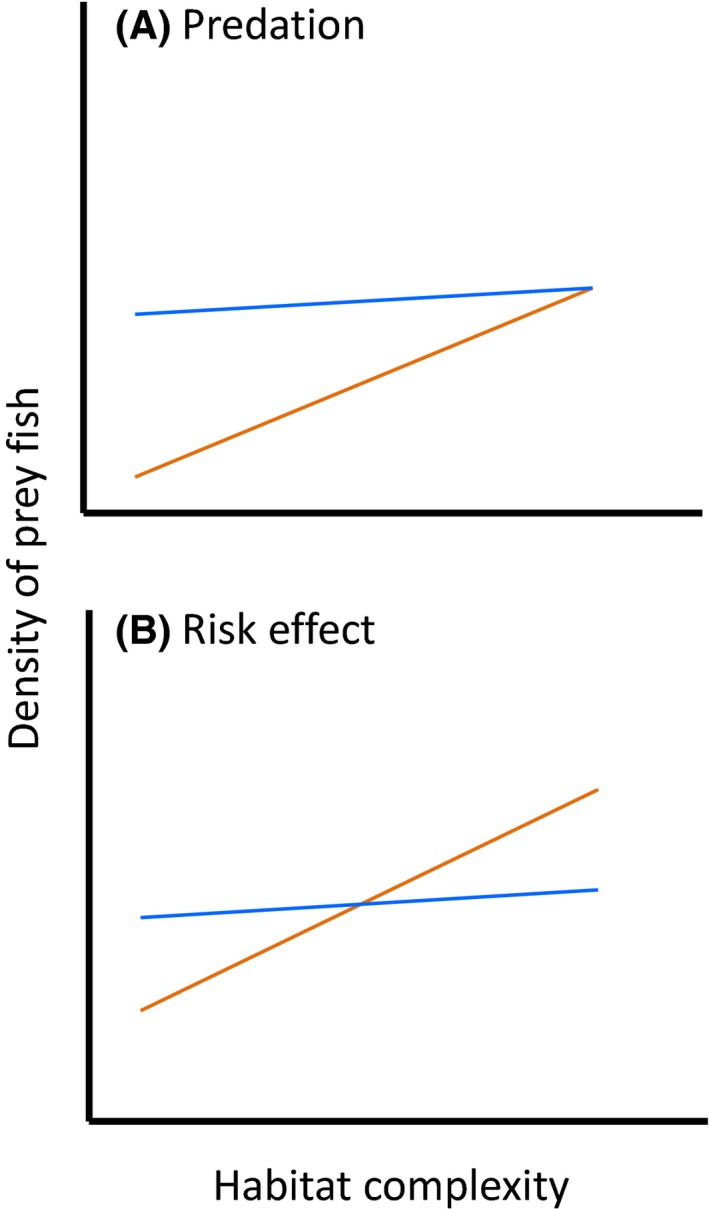
Predicted interactive effects of marine reserves (where a generalist predator is more abundant) and habitat complexity (where more complex habitat provides greater refuge from predation) on the densities of prey fish, under two different models. The two lines represent prey densities inside (in orange) and outside (in blue) marine reserves. If greater predation is occurring in reserves (A), a main effect of reserve status is expected, such that overall mean densities are lower inside reserves predation rates are greater inside reserves. Stronger positive associations with habitat complexity are expected due to prey being particularly vulnerable to predation in low‐complexity habitats, and/or a risk effect, where prey more strongly prefer complex habitats. Under the risk effect‐only model (B), predation is no greater inside reserves so there is no main effect of reserves on densities of prey—the overall heights of the two lines are similar. In this case, any stronger positive relationship between prey and habitat complexity is likely due to risk effects. Another possibility (not shown here) is that neither overall densities of prey (main effect of reserves) nor their relationships with habitat complexity (reserve–habitat interaction) are affected by reserves

If greater predation rates were occurring inside versus outside reserves, we would expect to see a density‐mediated effect where overall densities of small fishes are lower inside versus outside reserves (i.e., a main effect of reserves). A reserve–habitat interaction might also arise directly through predation being disproportionately high in low‐complexity habitats inside reserves and/or through risk effects (Fig. 2A). However, any potential increases in predation rates in reserves might be mitigated or even eliminated by risk effects, particularly if, like snapper, the predators are generalists that can target easier prey. We consider that, where there is no evidence of greater predation occurring inside reserves (i.e., no main effects of reserves), any stronger associations between densities of small fishes and habitat complexity (i.e., reserve–habitat interactions) observed are likely to be due to risk effects only (Fig. 2B). If neither risk effects nor predation rates were influenced by enhanced densities of predators in reserves, we would expect to see neither a main effect of reserves nor a reserve–habitat interaction.

Here, we surveyed assemblages of small benthic reef fishes and the occurrence of habitat features in and around three marine reserves in northern NZ over 3 years. We used Bayesian univariate and multivariate generalized linear mixed models to simultaneously evaluate support for the effects of marine reserve status, habitat features, reserve–habitat interactions, and several other factors inherent in the experimental design of the study, on the assemblage structure, species richness, and the densities of small benthic reef fishes. Specifically, we evaluated support for the predictions that (1) when controlling for habitat, assemblages of small benthic fishes would be different inside versus outside marine reserves, with some species having lower densities in reserves due to greater predation pressure, (2) assemblages of small fishes change along gradients in habitat and, in particular, densities of some species would be greater in more complex habitat where refuges are more available, and (3) an interaction between reserve status and habitat complexity would occur, where greater densities of predators within reserves give rise to stronger associations between habitat features and assemblages of prey fishes.

## 2 Materials and Methods

### 2.1 Experimental design and data collection

**Figure 3:**
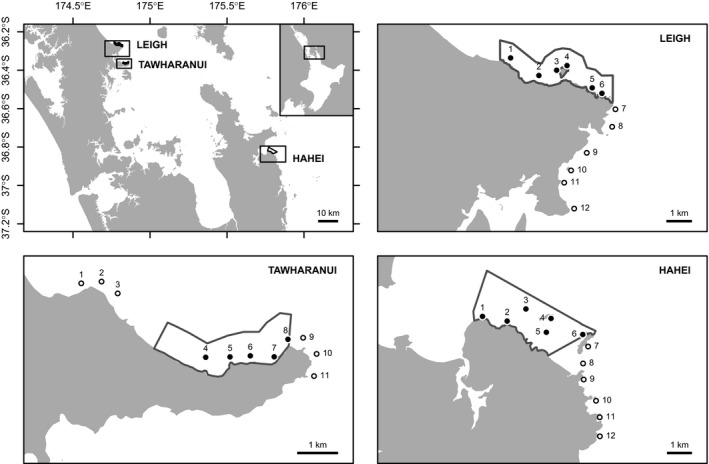
Map of the three locations and 35 sites (solid and open circles for reserve and nonreserve sites, respectively)

Surveys of benthic reef fishes were undertaken inside and outside marine reserves at three locations in northeast New Zealand (namely, Leigh, Tāwharanui, and Hahei; Fig. 3) in a structured, hierarchical design. Sampling was repeated between February and May in each of 3 years, 2011–2013. No survey was carried out at Tāwharanui in 2013, however, resulting in a total of 16 surveys. Surveys (and therefore inferences) were restricted to kelp‐forest habitat at a target depth of 10 m (± 2 m for 94% of data; all within ± 4 m). Kelp forest was chosen because Willis and Anderson (2003) reported the strongest effects of a marine reserve on densities of small fishes in this habitat, and this depth range was targeted because it is occupied by a range of triplefin species (e.g., Wellenreuther et al., 2007) and is beyond the normal depth ranges of the predator–urchin–kelp trophic cascades observed in these reserves (Shears et al., 2008). At each location, the marine reserve and adjacent nonreserve coasts were divided into six areas of roughly 1 km in length (except for Tāwharanui, where there were only five areas inside the reserve due to a limited amount of reef at the target depth). One site was selected randomly within each area from known rocky reefs along the 10‐m‐depth contour, using a GIS‐based method in R (R Development Core Team 2014). Specifically, within each area, the intersection of rocky reef habitat with the 10‐m‐depth contour line was transformed into a series of points and one point was drawn at random to provide the geographic coordinates for the site to be sampled within that area. This ensured that valid inferences could be drawn for the targeted habitat and depth across all locations. The spatial positions of sites were kept consistent across years, although individual transects sampled within each site varied from year to year. All sites had similar broad‐scale environmental conditions, such as exposure and temperature.

At each site, two to four scuba divers surveyed a total of *n *=* *8 transects, each measuring 1 m wide by 5 m long. For each transect, a measuring tape was laid on a roughly horizontal area of reef prior to surveying, with the ends of the tape fixed at least 2 m from the ends of the transect to minimize disturbance to the fish. Transects were comprised of five individually surveyed contiguous 1 m by 1 m quadrats, with the widths delineated visually by estimating a distance of 50 cm either side of the tape. In each quadrat, the diver first visually searched and recorded counts for all benthic fishes, then recorded the presence or absence of a set of predefined habitat features (listed in Table S1 in Appendix S1). Cracks, crevices, and caves were searched for fish with the aid of a torch. One common species of triplefin, *Forsterygion maryannae*, was excluded from analyses because, unlike other triplefins, it usually schools in the water column, often above the kelp canopy. A total of 752 transects (3,760 m^2^) were surveyed.

### 2.2 Statistical modeling

For all analyses, the data were summed within transects, yielding a density for each species of fish per 5 m^2^, and a score from zero to five for each habitat feature, according to the number of quadrats in which the feature was observed. A principal component analysis (PCA) of the habitat scores reduced the dimensionality of the habitat information and identified important gradients in habitat structure (see Appendix S1 for more details). Three PCA axes were derived and interpreted as follows: Hab1 represented overall complexity; Hab2 represented the position along a gradient from the reef–sand boundary to the inner reef; and Hab3 represented the verticality of the bedrock. Hab1–Hab3 were used as predictor variables, along with the factors inherent in the experimental design of the study, in the models described below.

We fit three GLMMs to model, in turn, (1) species richness, (2) total fish density summed across all species, and (3) the counts of the nine most common species at the transect level (the remaining species were considered too rare to be modeled effectively, although they were included in distance‐based analyses; see Appendix S2). For model (3), all nine species were fit simultaneously within a single, multiple‐response model. GLMMs were fit using the MCMCglmm package (Hadfield, 2010) for R (R Development Core Team 2014). For model (1), a Gaussian error distribution was used. For models (2) and (3), we assumed a Poisson error distribution with a log‐link function for the linear predictor. Models (2) and (3) included observation‐level (i.e., transect‐level) random effects, resulting in Poisson log‐normal models (Aitchison & Ho, 1989) to allow for overdispersion in the counts (Elston, Moss, Boulinier, Arrowsmith, & Lambin, 2001).

The available base predictor variables (listed in full in Table S2, Appendix S2) included the categorical factors arising from the experimental design (namely Reserve status, Location, Year, Site, and all interactions) plus four continuous covariables, namely, Depth, Hab1, Hab2, and Hab3 (see Appendix S1). Interactions between the habitat axes and some broad‐scale spatial factors (namely Reserve status, Location, and the Reserve × Location interaction) were also included to test hypotheses regarding potential spatial variation in the effects of habitat. The factors Reserve, Location, and Year were all treated as fixed, while Site (nested in Reserve × Location) and its interactions were treated as random. The predictor variables were the same for all three models, except that Depth was dropped from models (1) and (2) because the posterior distributions of the slopes were centered near zero and, while individual species have different depth ranges, there was no reason to suspect an effect of Depth on species richness or total density for the range of depths investigated here. In addition to the base set of predictors, model (3) included a fixed factor for Species (with nine levels), as well as: (1) fixed interactions between Species and each of the fixed effects for Reserve, Location, Year, and the three habitat axes; and (2) random interactions for Species × Site, Species × Site × Year, and Species × Transect. For the random interactions involving Species, we fit unstructured variance–covariance matrices (using the “us” function from the MCMCglmm package, Hadfield, 2010) to allow for nonzero correlations among Species at the level of Sites, Site–Year combinations, and Transects.

Samples from the joint posterior distributions of all fitted parameters were obtained via Markov chain Monte Carlo (MCMC). For the models of species richness and total fish density, the MCMCs were run for a total of 13,000 iterations with a thinning rate of 1/10, from which a burn‐in of 3,000 was discarded. For the multispecies model, 1,100,000 iterations were run with a thinning rate of 1/1,000 and a burn‐in of 100,000. This provided a posterior sample of 1,000 for each of the three models. Convergence of the main parameters of interest was tested by observing the posterior traces and checking the effective sample sizes for parameters (Gelman et al., 2014). For the coefficients associated with all fixed effects, we used the default, uninformative normal prior distributions with mean zero and variance 1e^+10^. Following Gelman (2006), scaled, noncentral *F*‐distributions were used as priors for the variance components for the random effects, with the scale parameter set to 1 for species richness and to 0.3 for the total number of fish. The code used to fit these models is provided as Supporting Information S4.

A set of quantities of interest were calculated from the fitted GLMM parameters for each response variable (species richness, total fish density, and densities of each of the nine fish species) for each iteration of the MCMC. The posterior distributions of each quantity of interest were then summarized with a point estimate (median) and a 95% credible interval (CI, given by the 0.025 and 0.975 quantiles). If the CI for a quantity of interest did not contain zero, this was considered as support for the hypothesis that the effect was nonzero. The quantities of interest were estimated median values of the response variables, or some function of medians that represented an effect size, calculated at various levels of marginalization. For the Poisson lognormal models (2) and (3), medians of the responses on their original scales were obtained by exponentiating means estimated on the log scale. For model (1), population medians and means are equivalent because the identity link function was used.

**Figure 4:**
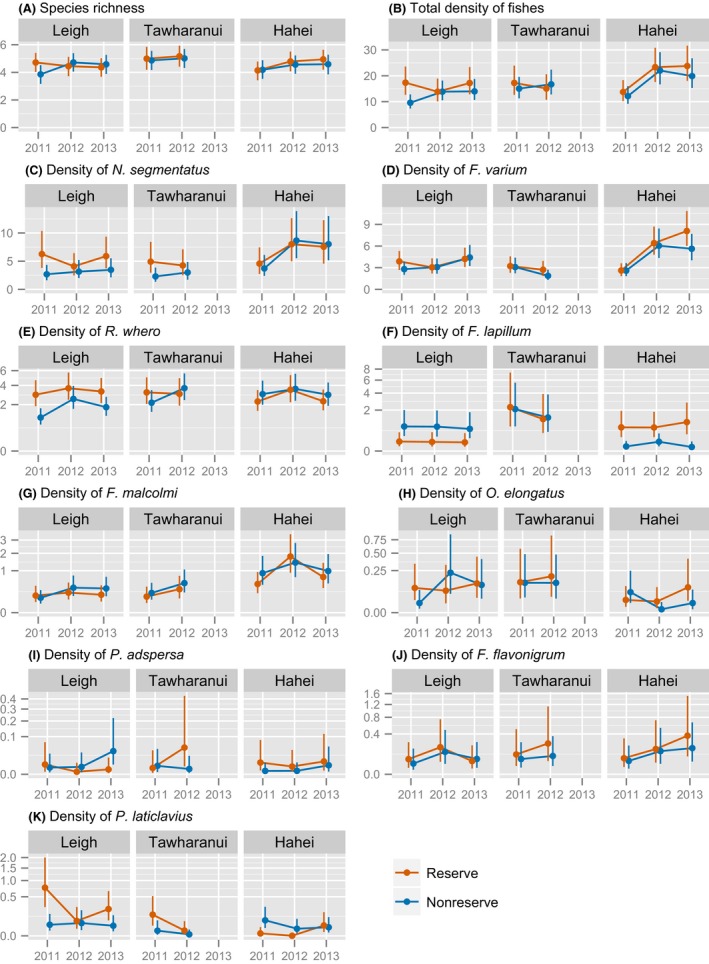
Estimated median densities per 5‐m^2^ transect for each combination of Location, Reserve status, and Year, with 95% credible intervals, for (A) species richness, (B) total number of fish, and (C–K) each of the nine most common species. Estimates are for a standardized median habitat at a depth of 10 m. In some cases, the y‐axes for (E–K) are shown on a square root scale for clarity

**Figure 5:**
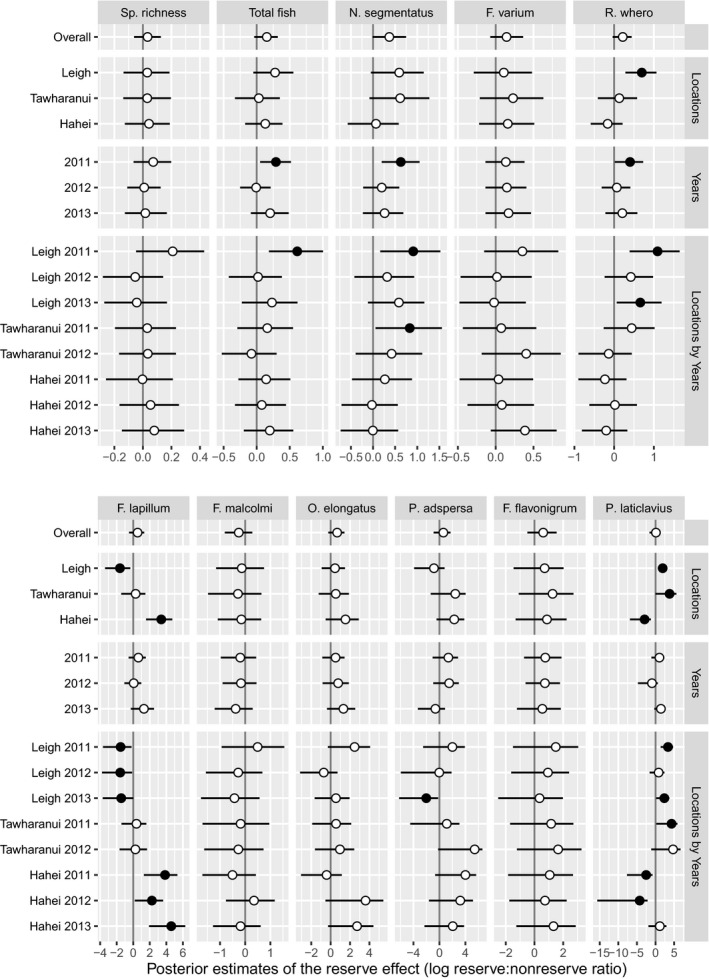
Estimated main effect of marine reserves on species richness and densities of small benthic reef fishes (means and 95% credible intervals of the log ratio of reserve versus nonreserve medians) shown for the overall study (i.e., averaged across all Location–Year combinations), for each Location (averaged across Years), and for each Year (averaged across Locations). Filled circles indicate cases where the 95% credible interval does not contain zero. Estimates are standardized for habitat and depth using Bayesian hierarchical models

**Figure 6:**
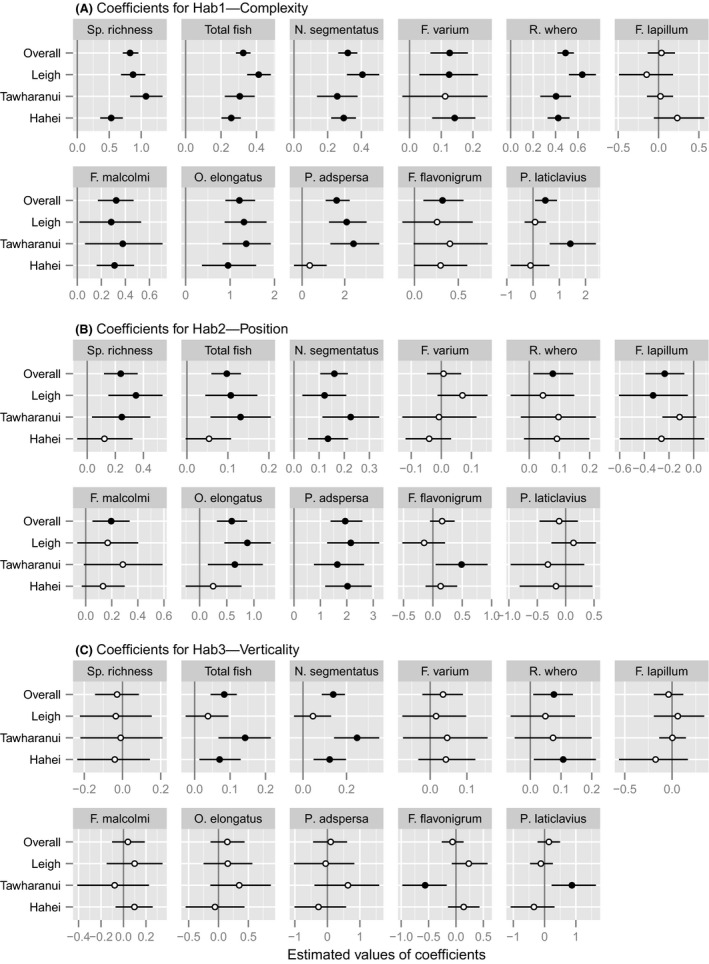
Main effects of 3 habitat gradients on species richness and densities of small benthic reef fishes (shown as means and 95% CIs of the coefficients associated with each of 3 habitat axes) estimated for the overall study and for each Location. The axes are (A) Hab1, overall habitat complexity; (B) Hab2, position from reef–sand edge to inner reef; and (C) Hab3, bedrock verticality. Filled circles indicate cases where the CI does not contain zero

**Figure 7:**
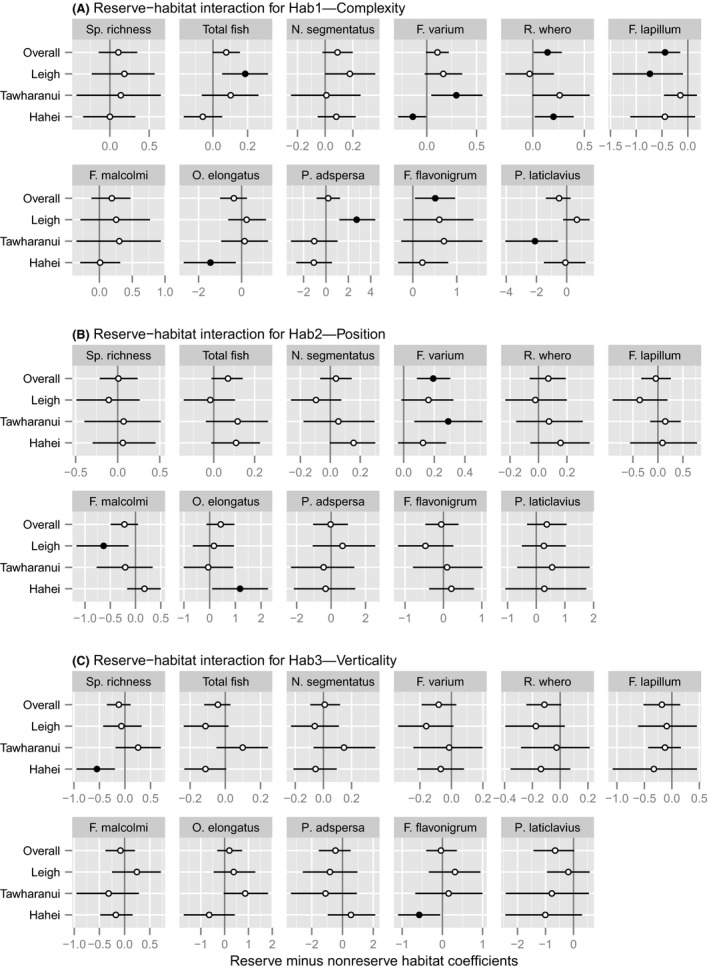
Effects of marine reserves on habitat associations (i.e., reserve‐habitat interactions, shown as means and 95% CIs of the differences between habitat coefficients inside versus outside reserves) in species richness and densities of small benthic reef fishes, estimated for the overall study and for each Location. Filled circles indicated cases where the CI does not contain zero. See caption for Fig. 6 for interpretations of the habitat axes

The quantities of interest were as follows: (1) the median values of the response variables for each of the 16 surveys, that is, for each combination of Reserve status, Location, and Year (Fig. 4); (2) the effect size for the main effect of Reserve status, calculated as the log of the ratio of reserve versus nonreserve medians (Fig. 5); (3) the main effects of habitat—the estimated values of coefficients (slopes) associated with each of Hab1, Hab2, and Hab3 (Fig. 6; see also Appendix S1); and (4) Reserve–Habitat interactions, evaluated as the difference between estimated coefficients for Hab1, Hab2, and Hab3 inside versus outside reserves (Fig. 7). The nature of the nonzero interactions identified was further explored by estimating and plotting fitted median densities across the full range of values of the Habitat variable, separately for sites inside or outside reserves. Medians were estimated for a Depth of 10 m and, for (1) and (2), a score of zero for each of the three Habitat axes, which represented a standardized median habitat. Thus, the estimates are adjusted (or “controlled”) for any differences in Depth or Habitat among the surveys. Estimates of quantities of interest across species, years, and locations were plotted using the R library *ggplot2* (Wickham, 2009). Additional analyses of the habitat variables and distance‐based multivariate models of assemblage structure are available in Appendices S1 and S2, respectively. Full details of the taxa are available in Appendix S3.

The predictions from the two theoretical models (i.e., predation versus risk‐effect mitigation; Fig. 2) rely on the assumption that any differences between the observed patterns found inside versus outside reserves are due to greater densities of predators. A recent study of the same reserves estimated that densities of large snapper were, on average, 13.4 times greater inside versus outside reserves, although the effect size varied among locations (Leigh, 19.3; Tāwharanui 7.8; Hahei 16.0; Smith et al., 2014). Other potential sources of variation in densities of small fishes include larval supply, the availability of food (primarily microinvertebrates), or densities of predators other than snapper. Larval supply is unlikely to differ inside versus outside these reserves, due to tidal currents regularly moving water back and forth along these coasts (e.g., Le Port, Montgomery, & Croucher, 2014). We see no reason to suspect differences in food availability and predators as surveys were standardized to a single broad habitat type (kelp forest around 10 m depth). Furthermore, the inclusion of three replicate locations in this study, and considering only patterns that were common across locations, reduces the risk of there being any unknown important differences with reserve status.

## 3 Results

There were few consistent patterns apparent in the estimated medians inside versus outside reserves among locations and years for species richness, total fish density, or the densities of any of the individual species (Fig. 4). Accordingly, the GLMMs provided no strong statistical support for consistent, overall effects of marine reserves (Fig. 5). Mean species richness varied between four and five species per 5‐m transect, appearing slightly higher at Tāwharanui than the other two locations (Fig. 4A). The median total fish density per transect (ranging from 10 to 24) showed similar spatiotemporal patterns to species richness, although it was considerably higher in 2012–13 at Hahei, apparently due to higher numbers of *Notoclinops segmentatus* and *F. varium* (Fig. 4). In the survey at Leigh in 2011, the median total fish density was greater inside versus outside the reserve, yielding a nonzero reserve effect in 2011 at Leigh, and in 2011 overall (Fig. 5), seemingly due to increased densities of *N. segmentatus*,* Ruanoho whero*, and *Parablennius laticlavius*. There was marginal support for an overall reserve effect (Fig. 5) on *N. segmentatus*, with densities estimated to be 1.446 (95% CI: 0.993–2.111) times greater inside versus outside reserves, on average. This was primarily driven by high densities within reserves at Leigh and Tāwharanui in 2011 (Fig. 4C). There were some nonzero differences with reserve status at some locations and in some years (Fig. 5). Densities of *R. whero* were greater inside than outside the reserve at Leigh in two of the 3 years. In contrast, densities of *F. lapillum* were greater outside the reserve at Leigh but inside the reserve at Hahei. *P. laticlavius* had greater densities, on average, inside reserves at Leigh and Tāwharanui but outside the reserve at Hahei.

The main effects of habitat were generally strong. Species richness, total fish, and the densities of all but one species, namely *F. lapillum*, increased with increased habitat complexity (Hab1, Fig. 6A). In most cases, the strength of the effect varied to some extent among locations. Transects on broken, sandy habitat near the edges of reefs (i.e., lower values of Hab2) had, on average, fewer species and total fish than those on less sandy transects with closed *Ecklonia* canopy (higher values; Fig. 6B). Likewise, five of the nine species also showed positive relationships with Hab2, while *F. varium*,* F. flavonigrum*, and *P. laticlavius* showed no overall relationship, and *F. lapillum* had a negative relationship. Total fish density and densities of *N. segmentatus* and *R. whero* were positively associated with verticality (Hab3, Fig. 6C).

**Figure 8:**
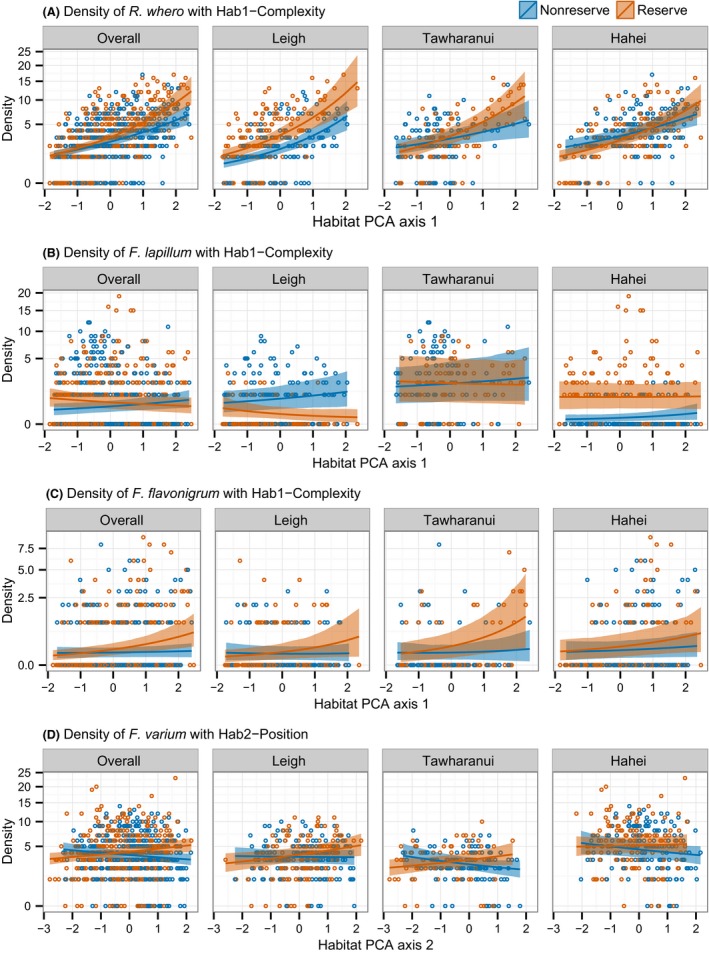
Reserve–Habitat interactions for 4 individual species of small benthic reef fish. Median densities (i.e., the exponential of the mean on the log scale) are shown across the range of habitat values, estimated separately inside and outside reserves, for the overall study and for each Location. Hab1 represents a gradient of increasing complexity, and Hab2 represents a gradient of sandy broken‐up reef to solid reef with closed *Eckonia* canopy. The *y*‐axes are shown on a square root scale for clarity

Reserve–habitat interactions, consistent across locations, were identified for four species: *R. whero*,* F. lapillum*, and *F. flavonigrum* with Hab1 (Fig. 7A), and *F. varium* with Hab2 (Fig. 7B). No such interactions with Hab3 were evident (Fig. 7C). On average, densities of *R. whero* (Fig. 8A) and *F. flavonigrum* (Fig. 8C) were estimated to be greater in more complex, feature‐rich habitats (i.e., higher Hab1 scores) both inside and outside reserves, but the relationships with Hab1 were stronger inside reserves. In general, higher densities occurred in more complex habitats inside reserves compared to similarly complex habitats outside reserves. Densities of *F. lapillum* were negatively associated with complexity inside reserves but positively associated with complexity outside reserves (Fig. 8B), although there was some variation in these relationships among locations. Associations between *F. varium* and Hab2 differed for reserves versus nonreserves (Fig. 8D); at all three locations, densities were higher, on average, on inner‐reef transects than those at the edges of reefs inside reserves while, outside reserves, the opposite pattern occurred. There was no strong evidence for an overall reserve–habitat interaction for either species richness or total fish density.

## 4 Discussion

We found no evidence for any overall main effects of marine reserves on assemblage structure, species richness, or densities of individual species of small benthic reef fish. There were some differences in assemblages observed between reserve and nonreserve areas, but strong interactions with location and year suggest that these patterns reflect more general spatial and temporal variations rather than any systematic effect of marine reserve protection.

These results contrast with those of Willis and Anderson (2003), who reported lower densities of some species inside versus outside the reserve at Leigh. There are several potential reasons for this discrepancy, one being that the two studies used different survey methods. The previous study used 3 m by 3 m caged rotenone stations. This lethal and relatively labor‐intensive method allowed the study of small cryptic species that are not reliably detected using visual methods (Willis 2001). For example, the clingfish *Dellichthys morelandi* (<7 cm, Francis, 2012) was found in significantly greater numbers outside versus inside the reserve (Willis & Anderson, 2003)—*D. morelandi* is strongly associated with the urchin *Evechinus chloroticus* (Dix, 1969), the density of which is known to be reduced by large snapper and lobster inside marine reserves (Babcock et al., 1999). Willis and Anderson (2003) also found significantly lower densities of three common species of triplefin (*F. lapillum*,* F. varium*, and *R. whero*), the total number of cryptic fish, and the species richness of cryptic fishes in kelp‐forest habitat inside versus outside reserves. In contrast, no such effects were found in the present study. In fact, while effect sizes of zero were plausible, the posterior densities for reserve effects were predominantly positive rather than negative for most species (Fig. 5). We consider that the inferences provided by the previous study were limited by a lack of spatial and temporal replication. Indeed, if we considered only the data from Leigh in the first year of the present study, we would have reported higher species richness and densities of small benthic fishes within the reserve (Figs 4A and 5). This highlights the need for empirical evaluations of the effects of reserves to be done across multiple years and multiple locations (Willis, Millar, Babcock, & Tolimieri, 2003), particularly when no data are available for time periods prior to the establishment of marine reserve protection.

The lack of main effects of reserves on densities of most species of small benthic fish in kelp forests is perhaps not surprising when one considers the potential mechanisms. For *D. morelandi*, there is a compelling mechanism due to a very strong association with a species known to be reduced in abundance by large snapper and lobster inside reserves. For the majority of fishes, however, an indirect effect of reserves via predation by snapper would seem unlikely, given that snapper are generalist predators and fish do not comprise a major part of the diet of reef‐based snapper in this region (estimated at 3.3% of total volume, Russell, 1983). Thus, their net trophic effect is likely weak for any individual taxon, particularly fishes, which are more mobile than invertebrate prey. On the other hand, blue cod (*Parapercis colias*, Pinguipedidae) is another fishery‐targeted predatory fish also known to benefit from marine reserves in New Zealand (Cole & Keuskamp, 1998; Pande et al., 2008). Blue cod consume fish to a greater extent than do snapper (40%, Russell, 1983) and thus, where abundant, may influence the densities of small fishes. However, blue cod are relatively rare in northern New Zealand (Smith, Duffy, & Leathwick, 2013) so are unlikely to exert significant trophic pressure in the reserves examined here.

Marine reserves could indirectly affect assemblages of small benthic fishes through restoration of kelp‐forest habitat via the predator–urchin–kelp trophic cascade (Babcock et al., 1999). If kelp forests support different fish assemblages than urchin‐grazed habitats, marine reserves would have broad‐scale indirect effects on small fishes, as has been shown for some invertebrate taxa (Shears & Babcock, 2003). Broad‐scale effects across multiple habitats across multiple reserves remain to be investigated; here, we focused on finer‐scale habitat associations within kelp forests at depths of c. 10 m, which is deeper than the predator–urchin–kelp trophic cascade normally occurs (Shears et al., 2008).

Our prediction of stronger positive associations between density and habitat complexity (represented by PCA axis Hab1) inside versus outside reserves was realized for *R. whero* and *F. flavonigrum* (Fig. 8A,C). We consider that this result, combined with no evidence for overall differences in density in reserves, is consistent with a risk effect whereby the fish are more strongly attracted to habitats that provide greater refuge in areas where they detect large numbers of predators (Fig. 2B). Reserve–habitat interactions were also observed for *F. lapillum* and *F. varium* (Fig. 8D), but were qualitatively different to the prediction made in Fig. 2B. The association between densities of *F. lapillum* and habitat complexity was positive outside reserves but negative inside reserves (Fig. 8B). This could be due, in part, to *R. whero* or *F. flavonigrum* competitively excluding *F. lapillum* in more complex habitat inside reserves; kelp forest at 10 m depth is not the typical habitat for *F. lapillum*, which is more often found in shallower water and in open habitats such as bare rock and cobbles (Francis, 2012). Finally, *F. varium* appeared to be more abundant on sandy broken reef at the sand–reef boundary (i.e., low values of Hab2) outside reserves, and on solid reef with closed *Ecklonia* canopy (i.e., high values of Hab2) inside reserves (Fig. 8D). Snapper forage at the sand–reef boundary (Ross, Thrush, Montgomery, Walker, & Parsons, 2007), and it may be that *F. varium* are avoiding high abundances of snapper at this boundary inside reserves, that is., a risk effect. Manipulative experiments would be required to test the specific mechanisms responsible for these observed patterns. Nonetheless, given the suspected ubiquity of risk effects in marine systems (Dill et al., 2003) and the lack of any observed main effect of reserves on overall density, we consider that risk effects provide likely explanations for the different habitat associations inside versus outside reserves observed here.

After decades of research, the full range of indirect effects of fishing, and protection from fishing, is still poorly understood. We consider that observational studies coupled with careful consideration of ecological mechanisms can improve our knowledge of the indirect effects of reserves on fishes. Many past studies have employed sampling methods designed to enumerate large, fishery‐targeted species (e.g., 25–50 m visual transects or video‐based surveys). Data on nontargeted species are generally collected as a secondary objective, and are often analyzed with little consideration of the mechanisms by which indirect effects might occur or the particular species that are likely to be affected. Moreover, broader scale sampling methods routinely omit small benthic species, which are often the target of predators and are thus most likely to be affected indirectly. There has been a narrow focus on consumptive effects (or “density‐mediated interactions,” Dill et al., 2003), which solely address the hypothesis that densities of prey fish are reduced by predators. Such effects can be mitigated or nullified by behavioral plasticity, such as prey‐switching by generalist predators (Jennings & Kaiser, 1998; Roberts, 1995) or predator‐avoidance risk effects in prey, as suggested here. To progress our knowledge of the indirect effects of marine reserves, we must move beyond simple evaluations of the densities of arbitrary groups of species sampled by a particular methodology. A more mechanistic focus is needed, beginning with specifically designed observational studies that test predictions drawn from suspected relationships with species known to benefit from reserves and, where possible, follow‐up experimental manipulations to isolate the suspected mechanisms.

## Conflict of Interest

None declared.

## References

[ece32406-bib-0001] Agardy, M. T. (1994). Advances in marine conservation: The role of marine protected areas. Trends in Ecology & Evolution, 9, 267–270. 21236850 10.1016/0169-5347(94)90297-6

[ece32406-bib-0002] Aitchison, J. , & Ho, C. H. (1989). The multivariate Poisson‐log normal distribution. Biometrika, 76, 643–653.

[ece32406-bib-0003] Babcock, R. C. , Kelly, S. , Shears, N. T. , Walker, J. W. , & Willis, T. J. (1999). Changes in community structure in temperate marine reserves. Marine Ecology Progress Series, 189, 125–134.

[ece32406-bib-0004] Babcock, R. C. , Shears, N. T. , Alcala, A. C. , Barrett, N. S. , Edgar, G. J. , Lafferty, K. D. , … Russ, G. R. (2010). Decadal trends in marine reserves reveal differential rates of change in direct and indirect effects. Proceedings of the National Academy of Sciences of the United States of America, 107, 18256–18261. 20176941 10.1073/pnas.0908012107 PMC2972978

[ece32406-bib-0005] Carr, M. H. , & Hixon, M. A. (1995). Predation effects on early post‐settlement survivorship of coral‐reef fishes. Marine Ecology Progress Series, 124, 31–42.

[ece32406-bib-0006] Claudet, J. , Guidetti, P. , Mouillot, D. , Shears, N. T. , & Micheli, F. (2011) Ecological effects of marine protected areas: Conservation, restoration and functioning In Claudel J. (Eds.), Marine protected areas: A multidisciplinary approach (pp. 377). Cambridge, UK: Cambridge University Press.

[ece32406-bib-0007] Claudet, J. , Osenberg, C. , Domenici, P. , Badalamenti, F. , Milazzo, M. , Falcón, J. , … Planes, S. (2010). Marine reserves: Fish life history and ecological traits matter. Ecological Applications, 20, 830–839. 20437967 10.1890/08-2131.1

[ece32406-bib-0008] Cole, R. G. , & Keuskamp, D. (1998). Indirect effects of protection from exploitation: Patterns from populations of *Evechinus chloroticus* (Echinoidea) in northeastern New Zealand. Marine Ecology Progress Series, 173, 215–226.

[ece32406-bib-0009] Connell, S. D. , & Jones, G. P. (1991). The influence of habitat complexity on postrecruitment processes in a temperate reef fish population. Journal of Experimental Marine Biology and Ecology, 151, 271–294.

[ece32406-bib-0010] Creel, S. , & Christianson, D. (2008). Relationships between direct predation and risk effects. Trends in Ecology & Evolution, 23, 194–201. 18308423 10.1016/j.tree.2007.12.004

[ece32406-bib-0011] Dill, L. M. , Heithaus, M. R. , & Walters, C. J. (2003). Behaviorally mediated indirect interactions in marine communities and their conservation implications. Ecology, 84, 1151–1157.

[ece32406-bib-0012] Dix, T. G. (1969). Association between the echinoid *Evechinus chloroticus* (Val.) and the clingfish *Dellichthys morelandi* Briggs. Pacific Science, 23, 332–336.

[ece32406-bib-0013] Edgar, G. J. , & Shaw, C. (1995). The production and trophic ecology of shallow‐water fish assemblages in southern Australia II. Diets of fishes and trophic relationships between fishes and benthos at Western Port, Victoria. Journal of Experimental Marine Biology and Ecology, 194, 83–106.

[ece32406-bib-0014] Edgar, G. J. , & Stuart‐Smith, R. D. (2009). Ecological effects of marine protected areas on rocky reef communities—a continental‐scale analysis. Marine Ecology Progress Series, 388, 51–62.

[ece32406-bib-0015] Edgar, G. J. , Stuart‐Smith, R. D. , Willis, T. J. , Kininmonth, S. , Baker, S. C. , Banks, S. , … Thomson, R. J. (2014). Global conservation outcomes depend on marine protected areas with five key features. Nature, 506, 216–220. 24499817 10.1038/nature13022

[ece32406-bib-0016] Elston, D. A. , Moss, R. , Boulinier, T. , Arrowsmith, C. , & Lambin, X. (2001). Analysis of aggregation, a worked example: Numbers of ticks on red grouse chicks. Parasitology, 122, 563–569. 11393830 10.1017/s0031182001007740

[ece32406-bib-0017] Feary, D. A. , & Clements, K. D. (2006). Habitat use by triplefin species (Tripterygiidae) on rocky reefs in New Zealand. Journal of Fish Biology, 69, 1031–1046.

[ece32406-bib-0018] Francis, M. P. (2012). Coastal fishes of New Zealand (4th ed.). Nelson, New Zealand: Craig Potton Publishing.

[ece32406-bib-0019] Game, E. T. , Grantham, H. S. , Hobday, A. J. , Pressey, R. L. , Lombard, A. T. , Beckley, L. E. , … Richardson, A. J. (2009). Pelagic protected areas: The missing dimension in ocean conservation. Trends in Ecology & Evolution, 24, 360–369. 19324450 10.1016/j.tree.2009.01.011

[ece32406-bib-0020] Gelman, A. (2006). Prior distributions for variance parameters in hierarchical models. Bayesian Analysis, 1, 1–19.

[ece32406-bib-0021] Gelman, A. , Carlin, J. B. , Stern, H. S. , Dunson, D. B. , Vehtari, A. , & Rubin, D. B. (2014). Bayesian data analysis (3rd ed.). Boca Raton, FL: CRC Press.

[ece32406-bib-0022] Godfriaux, B. L. (1969). Food of predatory demersal fish in Hauraki Gulf. New Zealand Journal of Marine and Freshwater Research, 3, 518–544.

[ece32406-bib-0023] Grabowski, J. H. (2004). Habitat complexity disrupts predator–prey interactions but not the trophic cascade on oyster reefs. Ecology, 85, 995–1004.

[ece32406-bib-0024] Graham, N. A. J. , Evans, R. D. , & Russ, G. R. (2003). The effects of marine reserve protection on the trophic relationships of reef fishes on the Great Barrier Reef. Environmental Conservation, 30, 200–208.

[ece32406-bib-0025] Guidetti, P. , Baiata, P. , Ballesteros, E. , Di Franco, A. , Hereu, B. , Macpherson, E. , … Sala, E. (2014). Large‐scale assessment of Mediterranean marine protected areas effects on fish assemblages. PLoS ONE, 9, e91841. 24740479 10.1371/journal.pone.0091841 PMC3989174

[ece32406-bib-0026] Hadfield, J. D. (2010). MCMC methods for multi‐response generalized linear mixed models: The MCMCglmm R package. Journal of Statistical Software, 33, 1–22. 20808728 PMC2929880

[ece32406-bib-0027] Heithaus, M. R. , Frid, A. , Wirsing, A. J. , & Worm, B. (2008). Predicting ecological consequences of marine top predator declines. Trends in Ecology & Evolution, 23, 202–210. 18308421 10.1016/j.tree.2008.01.003

[ece32406-bib-0028] Hixon, M. A. , & Jones, G. P. (2005). Competition, predation, and density‐dependent mortality in demersal marine fishes. Ecology, 86, 2847–2859.

[ece32406-bib-0029] Jennings, S. , Grandcourt, E. M. , & Polunin, N. V. C. (1995). The effects of fishing on the diversity, biomass and trophic structure of Seychelles’ reef fish communities. Coral Reefs, 14, 225–235.

[ece32406-bib-0030] Jennings, S. , & Kaiser, M. J. (1998). The effects of fishing on marine ecosystems. Advances in Marine Biology, 34, 352.

[ece32406-bib-0031] Langlois, T. J. , Harvey, E. S. , & Meeuwig, J. J. (2012). Strong direct and inconsistent indirect effects of fishing found using stereo‐video: Testing indicators from fisheries closures. Ecological Indicators, 23, 524–534.

[ece32406-bib-0032] Le Port, A. , Montgomery, J. C. , & Croucher, A. E. (2014). Biophysical modelling of snapper *Pagrus auratus* larval dispersal from a temperate MPA. Marine Ecology Progress Series, 515, 203–215.

[ece32406-bib-0033] Lester, S. E. , Halpern, B. S. , Grorud‐Colvert, K. , Lubchenco, J. , Ruttenberg, B. I. , Gaines, S. D. , … Warner, R. R. (2009). Biological effects within no‐take marine reserves: A global synthesis. Marine Ecology Progress Series, 384, 33–46.

[ece32406-bib-0034] Lima, S. L. , & Dill, L. M. (1990). Behavioral decisions made under the risk of predation: A review and prospectus. Canadian Journal of Zoology, 68, 619–640.

[ece32406-bib-0035] Ling, S. D. , Scheibling, R. E. , Rassweiler, A. , Johnson, C. R. , Shears, N. , Connell, S. D. , … Johnson, L. E. (2015). Global regime shift dynamics of catastrophic sea urchin overgrazing. Philosophical Transactions of the Royal Society B: Biological Sciences, 370, 20130269.

[ece32406-bib-0036] McClanahan, T. R. , Muthiga, N. A. , Kamukuru, A. T. , Machano, H. , & Kiambo, R. W. (1999). The effects of marine parks and fishing on coral reefs of northern Tanzania. Biological Conservation, 89, 161–182.

[ece32406-bib-0037] Micheli, F. , Benedetti‐Cecchi, L. , Gambaccini, S. , Bertocci, I. , Borsini, C. , Osio, G. C. , & Romano, F. (2005). Cascading human impacts, marine protected areas, and the structure of Mediterranean reef assemblages. Ecological Monographs, 75, 81–102.

[ece32406-bib-0038] Micheli, F. , Halpern, B. S. , Botsford, L. W. , & Warner, R. R. (2004). Trajectories and correlates of community change in no‐take marine reserves. Ecological Applications, 14, 1709–1723.

[ece32406-bib-0039] Molloy, P. P. , McLean, I. B. , & Côté, I. M. (2009). Effects of marine reserve age on fish populations: A global meta‐analysis. Journal of Applied Ecology, 46, 743–751.

[ece32406-bib-0040] Mosquera, I. , Côté, I. M. , Jennings, S. , & Reynolds, J. D. (2000). Conservation benefits of marine reserves for fish populations. Animal Conservation, 3, 321–332.

[ece32406-bib-0041] Pande, A. , MacDiarmid, A. B. , Smith, P. J. , Davidson, R. J. , Cole, R. G. , Freeman, D. , … Gardner, J. (2008). Marine reserves increase the abundance and size of blue cod and rock lobster. Marine Ecology Progress Series, 366, 147–158.

[ece32406-bib-0042] Peacor, S. D. , & Werner, E. E. (2001). The contribution of trait‐mediated indirect effects to the net effects of a predator. Proceedings of the National Academy of Sciences of the United States of America, 98, 3904–3908. 11259674 10.1073/pnas.071061998 PMC31151

[ece32406-bib-0043] Preisser, E. L. , Bolnick, D. I. , & Benard, M. F. (2005). Scared to death? The effects of intimidation and consumption in predator–prey interactions. Ecology, 86, 501–509.

[ece32406-bib-0044] R Development Core Team (2014). R: A language and environment for statistical computing. Vienna, Austria: R Foundation for Statistical Computing.

[ece32406-bib-0045] Roberts, C. M. (1995). Effects of fishing on the ecosystem structure of coral reefs. Conservation Biology, 9, 988–995. 10.1046/j.1523-1739.1995.9051332.x-i1 34261234

[ece32406-bib-0046] Ross, P. M. , Thrush, S. F. , Montgomery, J. C. , Walker, J. W. , & Parsons, D. M. (2007). Habitat complexity and predation risk determine juvenile snapper (*Pagrus auratus*) and goatfish (*Upeneichthys lineatus*) behaviour and distribution. Marine & Freshwater Research, 58, 1144–1151.

[ece32406-bib-0047] Russ, G. R. , & Alcala, A. C. (1998). Natural fishing experiments in marine reserves 1983‐1993: Community and trophic responses. Coral Reefs, 17, 383–397.

[ece32406-bib-0048] Russell, B. C. (1983). The food and feeding habits of rocky reef fish of north‐eastern New Zealand. New Zealand Journal of Marine and Freshwater Research, 17, 121–145.

[ece32406-bib-0049] Salomon, A. K. , Gaichas, S. K. , Shears, N. T. , Smith, J. E. , Madin, E. M. P. , & Gaines, S. D. (2010). Key features and context‐dependence of fishery‐induced trophic cascades. Conservation Biology, 24, 382–394. 20151987 10.1111/j.1523-1739.2009.01436.x

[ece32406-bib-0050] Schmitz, O. J. , Krivan, V. , & Ovadia, O. (2004). Trophic cascades: The primacy of trait‐mediated indirect interactions. Ecology Letters, 7, 153–163.

[ece32406-bib-0051] Shears, N. T. , & Babcock, R. C. (2003). Continuing trophic cascade effects after 25 years of no‐take marine reserve protection. Marine Ecology Progress Series, 246, 1–16.

[ece32406-bib-0052] Shears, N. T. , Babcock, R. C. , & Salomon, A. K. (2008). Context‐dependent effects of fishing: Variation in trophic cascades across environmental gradients. Ecological Applications, 18, 1860–1873. 19263884 10.1890/07-1776.1

[ece32406-bib-0053] Smith, A. N. H. , Anderson, M. J. , Millar, R. B. , & Willis, T. J. (2014). Effects of marine reserves in the context of spatial and temporal variation: An analysis using Bayesian zero‐inflated mixed models. Marine Ecology Progress Series, 499, 203–216.

[ece32406-bib-0054] Smith, A. N. H. , Duffy, C. A. J. , & Leathwick, J. R. (2013). Predicting the distribution and relative abundance of fishes on shallow subtidal reefs around New Zealand. Science for Conservation, 323, 25.

[ece32406-bib-0055] Syms, C. (1995). Multi‐scale analysis of habitat association in a guild of blennioid fishes. Marine Ecology Progress Series, 125, 31–43.

[ece32406-bib-0056] Tetreault, I. , & Ambrose, R. F. (2007). Temperate marine reserves enhance targeted but not untargeted fishes in multiple no‐take MPAs. Ecological Applications, 17, 2251–2267. 18213966 10.1890/06-0161.1

[ece32406-bib-0057] Wellenreuther, M. , Barrett, P. , & Clements, K. (2007). Ecological diversification in habitat use by subtidal triplefin fishes (Tripterygiidae). Marine Ecology Progress Series, 330, 235–246.

[ece32406-bib-0058] Wickham, H. (2009). ggplot2: Elegant graphics for data analysis. New York: Springer Science & Business Media.

[ece32406-bib-0059] Willis, T. J. , & Anderson, M. J. (2003). Structure of cryptic reef fish assemblages: Relationships with habitat characteristics and predator density. Marine Ecology Progress Series, 257, 209–221.

[ece32406-bib-0060] Willis, T. J. , Millar, R. B. , & Babcock, R. C. (2003). Protection of exploited fish in temperate regions: High density and biomass of snapper *Pagrus auratus* (Sparidae) in northern New Zealand marine reserves. Journal of Applied Ecology, 40, 214–227.

[ece32406-bib-0061] Willis, T. J. , Millar, R. B. , Babcock, R. C. , & Tolimieri, N. (2003). Burdens of evidence and the benefits of marine reserves: Putting descartes before des horse? Environmental Conservation, 30, 97–103.

[ece32406-bib-0101] Willis, T. J. (2001). Visual census methods underestimate density and diversity of cryptic reef fishes. Journal of Fish Biology, 59, 1408–1411.

