# Supplementary material for: Marine reserves indirectly affect fine‐scale habitat associations, but not overall densities, of small benthic fishes
Source: Ecol Evol. 2016 Aug 29;6(18):6648–61. doi: 10.1002/ece3.2406 (PMC5058535; doi:10.1002/ece3.2406)
Supplement: Supplementary file 1 [file ECE3-6-6648-s001.docx]

Appendix S1. Analysis of fine-scale habitat features

Table S1presents a list of the habitat variables recorded in each quadrat, and the axes obtained from a principle components analysis (PCA) of the habitat scores summed at the transect level. Three interpretable gradients were identified by the PCA of habitat variables which, together, accounted for 55% of the variation in habitat (Table A1). The first axis (Hab1) had positive loadings for all habitat variables, particularly Cobble, Boulder, Crack and Crevice, and can be interpreted as a measure of overall habitat ‘complexity’. Hab1 explained 30% of the variation in habitat variables, and correlated strongly with the total number of habitat features (Pearson’s *r* = 0.96). The second axis (Hab2) contrasted Sand and Gravel with closed canopy of the kelp *Ecklonia radiata* (Canopy) and Wall. Sand was sometimes observed in transects that were close to the reef-sand boundary. Thus, this axis was interpreted to represent a gradient from broken-up rock at the reef-sand boundary to the inner reef, where there is more continuous reef substratum, enabling kelp forests to form complete canopies. Thus, sites with low values of Hab2 are likely to be near the reef edge. This axis will be referred to as ‘position’. The third axis (Hab3) primarily contrasted Boulder and Canopy with Wall and Crack. Higher values of Hab3 likely indicate more vertical solid reef structures as opposed to horizontal base reef with boulders (i.e. ‘verticality’).

We tested for differences in mean depth and mean scores for three habitat gradients (Figure S1) inside *vs* outside reserves, for the overall study and for each of the three locations, using linear mixed models fit using the lme4::lmer() function in R. We included Site as a random factor, and Reserve and the Reserve-by-Location interaction as fixed factors. Significant differences were detected for mean depths and Hab2 at some locations, and for the overall study (Figure S1). These differences highlight the importance of taking these measures into account when estimating the effects of reserves.

Table S1. The habitat features (and definitions) for which presence or absence was recorded in each quadrat. Also provided here are the loadings (standardised coefficients) of each habitat feature for each of the first three principal component axes from a PCA of 12 habitat variables. Loadings that exceed 0.4 are shown in bold.

|  |  | PCA Axis Loadings | | |
| --- | --- | --- | --- | --- |
| Habitat feature | Definition | Hab1 | Hab2 | Hab3 |
| Platform | Horizontal rocky surface | 0.02 | 0.00 | -0.11 |
| Wall | Near-vertical rocky surface | 0.34 | 0.27 | **0.42** |
| Overhang | Near-horizontal overhanging rocky surface | 0.28 | 0.18 | -0.05 |
| Sand | Fine sand (< 0.5 cm dia) | 0.19 | **-0.59** | -0.21 |
| Gravel | Gravel (< 5 cm dia) | 0.20 | -0.38 | 0.00 |
| Cobble | Cobbles (5-20 cm dia) | 0.35 | -0.22 | -0.15 |
| Boulder | Rocks (> 20 cm dia) | **0.46** | -0.08 | **-0.45** |
| Crack | Fissure < 5 cm wide | 0.39 | 0.00 | 0.39 |
| Crevice | Fissure 5-20 cm wide | **0.43** | 0.14 | 0.20 |
| Cave | Fissure > 20 cm wide | 0.23 | 0.17 | -0.03 |
| Canopy | Closed canopy of *Ecklonia radiata* | 0.10 | **0.54** | **-0.59** |
| Sponge | Habitat-providing sponge (usually *Ancorina alata*) | 0.01 | 0.10 | 0.00 |
| Axis name |  | Complexity | Position | Verticality |
| Variation explained (%) |  | 29.8 | 14.8 | 10.9 |
| Cumulative variation explained (%) |  | 29.8 | 44.6 | 55.5 |


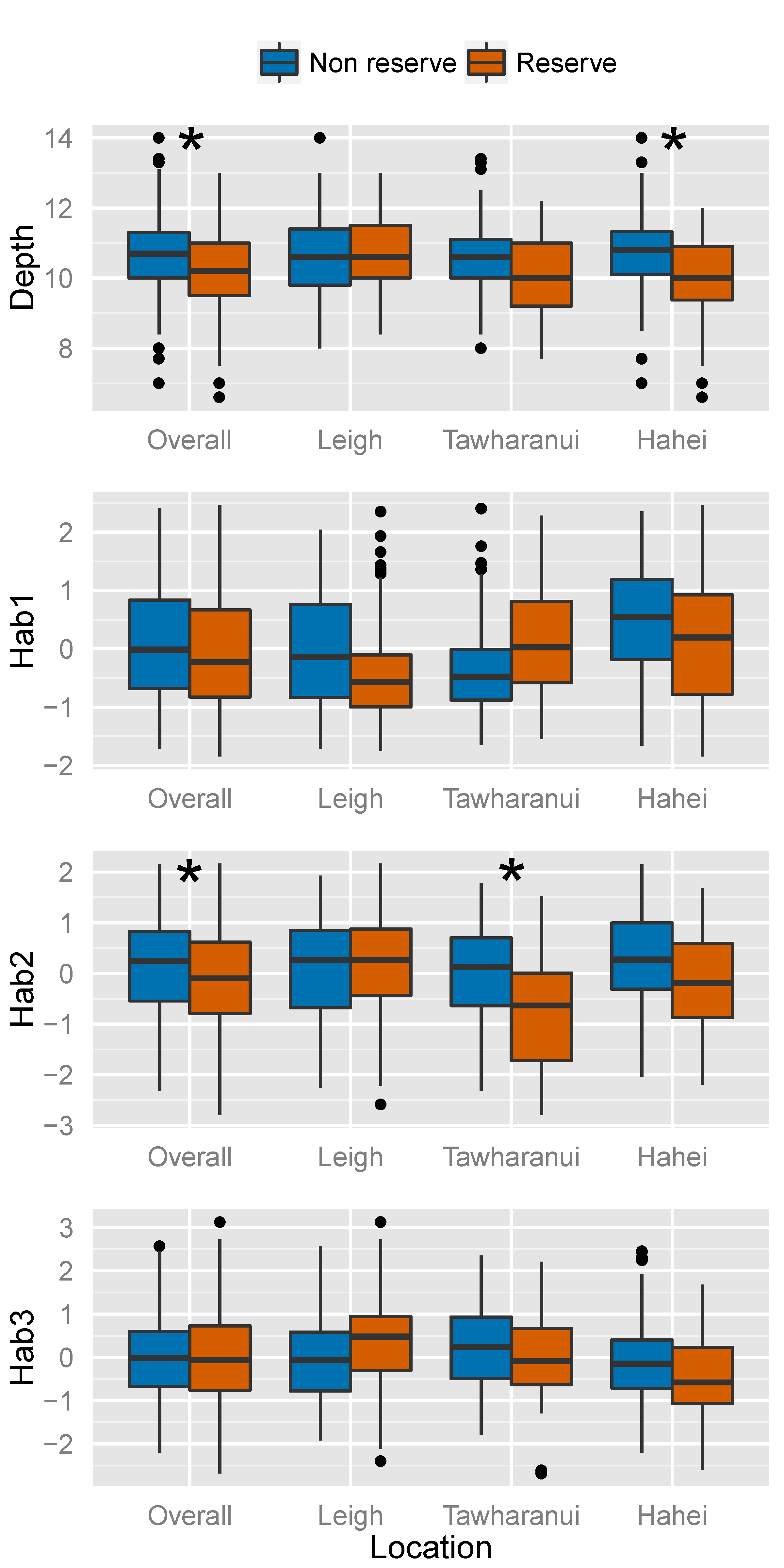


Figure S1. Boxplots showing the distribution of values of depth and the three PCA axes for habitat for reserve and non-reserve transects, for the overall study and at each location. An asterisk indicates a significant difference in the mean score inside *vs* outside the reserve (tested using mixed models with Site included as a random effect).
