# Supplementary material for: Marine reserves indirectly affect fine‐scale habitat associations, but not overall densities, of small benthic fishes
Source: Ecol Evol. 2016 Aug 29;6(18):6648–61. doi: 10.1002/ece3.2406 (PMC5058535; doi:10.1002/ece3.2406)
Supplement: Supplementary file 2 [file ECE3-6-6648-s002.docx]

Appendix S2. Distance-based multivariate analysis

A matrix of zero-adjusted Bray-Curtis dissimilarities (Clarke et al. 2006) was used to quantify ecological variation in assemblage structure. Partitioning of variation in assemblage structure on the basis of this matrix was done using permutational multivariate analysis of variance (PERMANOVA, Anderson 2001). The predictor variables (listed in full in Table S2) included categorical factors arising from the experimental design (namely Reserve status, Location, Year, Site, and all interactions) plus four continuous covariables: namely, Depth, Hab1, Hab2, and Hab3 (see Table 1 in article). Interactions between the habitat axes and some broad-scale spatial factors (namely Reserve status, Location, and the Reserve × Location interaction) were also included to test hypotheses regarding potential spatial variation in the effects of habitat. The factors Reserve, Location, and Year were all treated as fixed, while Site (nested in Reserve × Location) and its interactions were treated as random. Terms corresponding to fixed effects or interactions that were identified as significant by PERMANOVA were explored further using non-metric multi-dimensional scaling ordination (MDS) of distance matrices among appropriate centroids of combinations of factors of interest (see Figure S2). Calculations of distances among centroids (in the space of the original Bray-Curtis dissimilarity matrix), MDS ordinations, and PERMANOVA were implemented using the computer program PRIMER v6 (Clarke and Gorley 2006) with the PERMANOVA+ add-on (Anderson et al. 2008).

There was no significant main effect of Reserve status on fish assemblage structure (PERMANOVA, Table S2). Locations differed significantly, and an interaction between Reserve status and Location indicated that the reserve effect was not consistent across the three locations. For Leigh and Tāwharanui, the shift in community structure from assemblages inside to those outside the marine reserves occurs in a similar direction in the multivariate space (i.e., from upper to lower parts of the MDS plot); it occurs in the opposite direction for Hahei (Figure S2b). Assemblage structure was strongly associated with changes in Habitat, as represented by the three PCA axes (Table S2). The effects of two of these axes, namely Hab1 and Hab2, varied among Locations and inside *vs* outside reserves (see significant interactions with Location and Reserve-by-Location, Table S2). These results are in general agreement with those obtained using generalised linear mixed models presented in the main article.

Table S2. Permutational multivariate analysis of (co)variance (PERMANOVA) examining the effects of depth and habitat variables (as covariates) and factors in the structured sampling design on the structure of assemblages of benthic reef fish, based on adjusted Bray-Curtis distances of the transect-level abundances of all species. There were 635 residual *df*, and tests were based on Type III sums of squares and 999 permutations.

| **Source** | **df** | **Pseudo-*F*** | ***P*-value** |  |
| --- | --- | --- | --- | --- |
| Hab1 | 1 | 51.83 | 0.001 | *** |
| Hab2 | 1 | 10.41 | 0.001 | *** |
| Hab3 | 1 | 7.29 | 0.001 | *** |
| Hab1 × Res | 1 | 1.41 | 0.206 |  |
| Hab2 × Res | 1 | 1.66 | 0.132 |  |
| Hab3 × Res | 1 | 1.30 | 0.235 |  |
| Hab1 × Loc | 2 | 2.47 | 0.002 | ** |
| Hab2 × Loc | 2 | 1.98 | 0.026 | * |
| Hab3 × Loc | 2 | 1.47 | 0.113 |  |
| Hab1 × Res × Loc | 2 | 3.58 | 0.001 | *** |
| Hab2 × Res × Loc | 2 | 1.98 | 0.017 | * |
| Hab3 × Res × Loc | 2 | 0.99 | 0.471 |  |
| Depth | 1 | 2.67 | 0.009 | ** |
| Reserve | 1 | 1.51 | 0.193 |  |
| Location | 2 | 4.81 | 0.002 | ** |
| Year | 2 | 6.74 | 0.001 | *** |
| Res × Loc | 2 | 2.03 | 0.040 | * |
| Res × Year | 2 | 1.34 | 0.185 |  |
| Loc × Year | 3 | 5.38 | 0.001 | *** |
| Site (Loc × Res) | 29 | 6.55 | 0.001 | *** |
| Site (Loc × Res) × Year | 49 | 2.32 | 0.001 | *** |
| Res × Loc × Year | 3 | 1.46 | 0.104 |  |

Figure S2. Non-metric multidimensional scaling (MDS) plots of (a) the Site-by-Year centroids and (b) Location-by-Reserve-by-Year centroids, in Bray-Curtis space, shown by Location and Reserve status (R = reserve, NR = non-reserve). The 2-d stress for the MDS analyses were 0.13 (a) and 0.07 (b).
