# Supplementary material for: Marine reserves indirectly affect fine‐scale habitat associations, but not overall densities, of small benthic fishes
Source: Ecol Evol. 2016 Aug 29;6(18):6648–61. doi: 10.1002/ece3.2406 (PMC5058535; doi:10.1002/ece3.2406)
Supplement: Supplementary file 3 [file ECE3-6-6648-s003.docx]

Appendix S3. Taxa observed

Table S3. Taxa detected in the surveys, including the number of individuals of each taxon observed in the whole dataset. A total of 14,056 benthic reef fish were recorded from 23 taxa and 14 families. Ten of these taxa were triplefin species (Tripterygiidae), including the five species most often seen in the surveys. Around 75% of individuals belonged to the three most abundant species, *N. segmentatus*, *F. varium*, and *R. whero*. Most taxa were consistently identified to species, including all triplefins (TF). Moridae consisted mostly of the species *Pseudophycis breviuscula* and *Lotella rhacina*. Gobiesocidae were *Dellichthys morelandi* or *Gastrocyathus gracilis*. *Acanthoclinus* spp. were most likely *A. rua*, *A. marilynae*, or *A. littoreus*. The nine most abundant species (*)were modelled individually.

| Taxon | Common name | Family | No. observed |
| --- | --- | --- | --- |
| *Notoclinops segmentatus** | Blue-eyed TF | Tripterygiidae | 4503 |
| *Forsterygion varium** | Variable TF | Tripterygiidae | 3256 |
| *Ruanoho whero** | Spectacled TF | Tripterygiidae | 2753 |
| *Forsterygion lapillum** | Common TF | Tripterygiidae | 1026 |
| *Forsterygion malcolmi** | Banded TF | Tripterygiidae | 720 |
| *Optivus elongatus** | Slender roughy | Trachichthyidae | 550 |
| *Pempheris adspersa** | Bigeye | Pempheridae | 520 |
| *Forsterygion flavonigrum** | Yellow-black TF | Tripterygiidae | 366 |
| *Parablennius laticlavius** | Crested blenny | Blenniidae | 141 |
| *Scorpaena papillosa* | Dwarf scorpionfish | Scorpaenidae | 68 |
| Moridae | Morid cod | Moridae | 51 |
| *Acanthoclinus* spp. | Rockfish | Plesiopidae | 25 |
| *Karelepis stewarti* | Scaly-headed TF | Tripterygiidae | 18 |
| *Gymnothorax prasinus* | Yellow moray | Muraenidae | 16 |
| Gobiesocidae | Clingfish | Gobiesocidae | 10 |
| *Ruanoho decemdigitatus* | Long-finned TF | Tripterygiidae | 8 |
| *Conger verreauxi* | Conger eel | Congridae | 6 |
| *Gobiopsis atrata* | Black goby | Gobiidae | 6 |
| *Thalasseleotris iota* | Pygmy sleeper | Eleotridae | 5 |
| *Ericentrus rubrus* | Banded weedfish | Clinidae | 4 |
| *Notoclinops caerulepunctus* | Blue-dot TF | Tripterygiidae | 2 |
| *Cryptichthys jojettae* | Cryptic TF | Tripterygiidae | 1 |
| *Hypoplectrodes* sp. | Half-banded perch | Serranidae | 1 |
