# Supplementary material for: Marine reserves indirectly affect fine‐scale habitat associations, but not overall densities, of small benthic fishes
Source: Ecol Evol. 2016 Aug 29;6(18):6648–61. doi: 10.1002/ece3.2406 (PMC5058535; doi:10.1002/ece3.2406)
Supplement: Supplementary file 4 [file ECE3-6-6648-s004.zip › Supporting_Information_-_S4_-_Description_of_data_and_R_code_provided.docx]

Supporting Information S4:

Description of Data and R Code for Fitting Models using MCMCglmm

# **File list** Models.R

# FishData.Rdata

# **Description**

Supplement S4 provides data (*FishData.Rdata*) and code (*Models.R*) for applying generalised linear mixed models (GLMMs) to counts of small benthic fishes obtained from a hierarchically structured survey from inside and outside three marine reserves in north-eastern New Zealand. GLMMs were fit using the MCMCglmm package (Hadfield 2010) for R (R Development Core Team 2014). The file provided contains code to fit models to three response variables: (1) Species richness, (2) Total density of fishes, and (3) Multivariate counts of the nine most-common species, as described in the manuscript.

# **Literature Cited**

Hadfield, J. D. 2010. MCMC methods for multi-response generalized linear mixed models: the MCMCglmm R package. Journal of Statistical Software 33:1–22.

R Development Core Team. 2014. R: A Language and Environment for Statistical Computing. R Foundation for Statistical Computing, Vienna, Austria.
